# Asymptotic Bounds for the Time-Periodic Solutions to the Singularly Perturbed Ordinary Differential Equations

**DOI:** 10.1155/2013/301609

**Published:** 2013-12-04

**Authors:** Gabil M. Amiraliyev, Aysenur Ucar

**Affiliations:** Department of Mathematics, Sinop University, 57000 Sinop, Turkey

## Abstract

The periodical in time problem for singularly perturbed second order linear ordinary differential equation is considered. The boundary layer behavior of the solution and its first and second derivatives have been established. An example supporting the theoretical analysis is presented.

## 1. Introduction and Preliminaries

In this paper we investigate the equation

(1) $$\begin{array}{c} Lu\equiv \varepsilon u^{\prime \prime }+a\left(t\right)u^{\prime }+b\left(t\right)u=f\left(t\right),\;0<t<T, \end{array}$$

with the periodic conditions

(2) $$\begin{array}{c} u\left(0\right)=u\left(T\right),\;\;u^{\prime }\left(0\right)=u^{\prime }\left(T\right), \end{array}$$

where *ε* ∈ (0,1] is the perturbation parameter, 0 < *α* ≤ *a*(*t*) ≤ a*, 0 < *β* ≤ *b*(*t*) ≤ b*, and *f*(*t*) are the *T*-periodic functions satisfying *a*, *b*, *f* ∈ *C* ^1^[0, *T*].

Periodical in time problems arise in many areas of mathematical physics and fluid mechanics [1–3]. Various properties of periodical in time problems in the absence of boundary layers have been investigated earlier by many authors (see, e.g., [4, 5] and references therein).

The qualitative analysis of singular perturbation situations has always been far from trivial because of the boundary layer behavior of the solution. In singular perturbation cases, problems depend on a small parameter *ε* in such a way that the solution exhibits a multiscale character; that is, there are thin transition layers where the solution varies rapidly while away from layers and it behaves regularly and varies slowly [6–8].

We note that periodical in space variable problems and also their approximate solutions were investigated by many authors (see, e.g., [9–13]).

In this note we establish the boundary layer behaviour for *u*(*t*) of the solution of (1)-(2) and its first and second derivatives. The maximum principle, which is usually used for periodical boundary value problems, is not applicable here; because of this we use another approach which is convenient for this type of problems. The approach used here is similar to those in [9, 14, 15].

**Note 1**

Throughout the paper *C* denotes the generic positive constants independent of *ε*. Such a subscripted constant is also independent of *ε*, but its value is fixed.

**Lemma 1**

Let *δ*(*t*) ≥ 0 be the continuous function defined on [0, *T*] and *c*_0_(*t*), *ρ*(*t*) ∈ *C*[0, *T*] and *γ*, *μ* are given constants. If

(3) $$\begin{array}{c} \delta^{\prime }\left(t\right)+c_{0}\left(t\right)\delta \left(t\right)\leq \rho \left(t\right),\;\delta \left(0\right)\leq \mu \delta \left(T\right)+\gamma , \end{array}$$

then

(4) δ(t)≤(1−μe−∫0Tc0(s)ds)−1×(γe−∫0Tc0(η)dη+∫0Tρ(s)e−∫sTc0(η)dηds)e−∫0Tc0(η)dη+∫0tρ(s)e−∫sTc0(η)dηds

provided that

(5) $$\begin{array}{c} 1-\mu e^{-\int_{0}^{T}c_{0}(s)ds}>0. \end{array}$$

**Proof**

Inequality (4) can be easily obtained by using first order differential inequality containing initial condition.

## 2. Asymptotic Estimate

We now give a priori bounds on the solution and its derivatives for problem (1)-(2).

**Theorem 2**

The solution *u*(*t*) of the problem (1)-(2) satisfies the bound

(6) $$\begin{array}{c} \varepsilon {\left|u^{\prime }\right|}^{2}+{\left|u\right|}^{2}\leq C\int_{0}^{t}{\left|f\left(s\right)\right|}^{2}ds, \end{array}$$

provided that

(7) $$\begin{array}{c} \gamma =\lambda_{0}\;\underset{\left[0,T\right]}{\min}\left(2b\left(t\right)-a^{\prime }\left(t\right)\right)-\overset{-}{b^{*}}>0, \end{array}$$

where

(8) $$\begin{array}{c} \overset{-}{b^{*}}=\underset{\left[0,T\right]}{\max}\;b^{\prime }\left(t\right),\;\;0<\lambda_{0}<\frac{\left(\alpha +\sqrt{\alpha^{2}+8\beta }\right)}{4}. \end{array}$$

**Proof**

Consider the identity

(9) $$\begin{array}{c} Lu\left(u^{\prime }+\lambda u\right)=\left(u^{\prime }+\lambda u\right)f\left(t\right) \end{array}$$

with parameter *λ* > 0 which will be chosen later. By using the equalities

(10) $$\begin{array}{c} \varepsilon u^{\prime \prime }u^{\prime }=\frac{\varepsilon }{2}{\left[{\left(u^{\prime }\right)}^{2}\right]}^{\prime },\\ \lambda u^{\prime \prime }u=\lambda {\left(u^{\prime }u\right)}^{\prime }-\lambda {\left(u^{\prime }\right)}^{2},\\ \lambda a\left(t\right)u^{\prime }u=\frac{\lambda }{2}a\left(t\right){\left(u^{2}\right)}^{\prime }=\frac{\lambda }{2}{\left[a\left(t\right)u^{2}\right]}^{\prime }-\frac{\lambda }{2}a^{\prime }\left(t\right)u^{2},\\ b\left(t\right)u^{\prime }u=\frac{1}{2}b\left(t\right){\left(u^{2}\right)}^{\prime }=\frac{1}{2}{\left[b\left(t\right)u^{2}\right]}^{\prime }-\frac{1}{2}b^{\prime }\left(t\right)u^{2}, \end{array}$$

and the inequalities

(11) $$\begin{array}{c} u^{\prime }f\left(t\right)\leq \mu_{1}{\left(u^{\prime }\right)}^{2}+\frac{1}{4\mu_{1}}f^{2}\left(t\right),\;\mu_{1}>0,\\ \lambda uf\left(t\right)\leq \lambda \mu_{2}u^{2}+\frac{\lambda }{4\mu_{2}}f^{2}\left(t\right),\;\mu_{2}>0, \end{array}$$

in (9), we have

(12) {εu′2+2ελu′u+λa(t)u2+b(t)u2}′  ≤−2{a(t)−ελ−μ1}u′2   +{b′(t)+λa′(t)−2λb(t)+2λμ2}u2   +{12μ1+λ2μ2}f2(t).

Denoting now *δ*(*t*) = *εu* ^′2^ + 2*ελu*′*u* + *λa*(*t*)*u* ^2^ + *b*(*t*)*u* ^2^ and choosing *μ* = 1/2, we arrive at

(13) $$\begin{array}{c} \delta \left(t\right)\geq \frac{\varepsilon }{2}u^{\prime 2}+\left\{\beta +\lambda \left(\alpha -2\lambda \varepsilon \right)\right\}u^{2}. \end{array}$$

After taking $\lambda =\lambda_{0}<\left(\alpha +\sqrt{\alpha^{2}+8\beta }\right)/4$, the last inequality reduces to

(14) $$\begin{array}{c} \delta \left(t\right)\geq C_{0}\left(\varepsilon u^{\prime 2}+u^{2}\right), \end{array}$$

where

(15) $$\begin{array}{c} 0<C_{0}=\min\left\{\frac{1}{2},\beta +\lambda_{0}\left(\alpha -2\lambda_{0}\varepsilon \right)\right\}. \end{array}$$

On the other hand for the function *δ*(*t*) holds the following inequality clearly:

(16) δ(t)≤ε(1+λ)u′2+(b∗+ελ+λa∗)u2≤ε(1+λ0)u′2+(b∗+λ0+λ0a∗)u2.

For the right-hand side of inequality (12), we have

(17) 2{a(t)−ελ−μ1}u′2   +{−b′(t)−λa′(t)+2λb(t)−2λμ2}u2  ≥2ε{α−ελ0−μ1}u′2   +{−b∗−−λ0a′(t)+2λ0b(t)−2λ0μ2}u2  ≥αεu′2+γ2u2.

Taking into account *ε* ≤ 1 and *γ* > 0, after choosing *μ*_1_ = (*α* − 2*λ*_0_)/2 and *μ*_2_ = *γ*/4*λ*_0_, we have

(18) $$\begin{array}{c} \delta^{\prime }\left(t\right)\leq -C_{1}\delta \left(t\right)+\rho \left(t\right),\;\delta \left(0\right)=\delta \left(T\right), \end{array}$$

where

(19) $$\begin{array}{c} 0<C_{1}=\min\left\{\frac{\alpha }{1+\lambda_{0}},\frac{\gamma }{2\left(b^{*}+\lambda_{0}+\lambda_{0}a^{*}\right)}\right\},\\ \rho \left(t\right)=\left\{\frac{1}{2\mu_{1}}+\frac{\lambda }{2\mu_{2}}\right\}f^{2}\left(t\right). \end{array}$$

From (18) by using Lemma 1, we have

(20) $$\begin{array}{c} \delta \left(t\right)\leq \frac{e^{-C_{1}t}}{1-e^{-C_{1}T}}\int_{0}^{T}\rho \left(s\right)e^{-C_{1}(t-s)}ds+\int_{0}^{t}\rho \left(s\right)e^{-C_{1}(t-s)}ds \end{array}$$

which proves Theorem 2.

**Note 2**

As it is seen from (6)

(21) $$\begin{array}{c} \left|u\left(t\right)\right|\leq C{||f||}_{2}, \end{array}$$

where

(22) $$\begin{array}{c} {||f||}_{2}=\int_{0}^{T}{\left|f^{2}\left(s\right)\right|}^{1/2}ds. \end{array}$$

**Theorem 3**

Under the assumptions of Theorem 2, the following asymptotic estimates for the derivatives hold true:

(23) $$\begin{array}{c} \left|u^{\left(k\right)}\left(t\right)\right|\leq C\left\{1+\varepsilon^{1-k}e^{-\alpha t/\varepsilon }\right\},\;0\leq t\leq T,\;\;k=0,1,2. \end{array}$$

**Proof**

The case *k* = 0 directly follows from the identity (4).

For *k* = 1, the problem (1)-(2) can be rewritten as

(24) $$\begin{array}{c} \varepsilon u^{\prime \prime }+a\left(t\right)u^{\prime }=F\left(t\right),\;0<t<T, \end{array}$$

(25) $$\begin{array}{c} \left|u\left(0\right)\right|\leq C, \end{array}$$

(26) $$\begin{array}{c} u^{\prime }\left(0\right)=u^{\prime }\left(T\right), \end{array}$$

where

(27) $$\begin{array}{c} F\left(t\right)=f\left(t\right)-b\left(t\right)u \end{array}$$

and by virtue of Theorem 2

(28) $$\begin{array}{c} \left|F\left(t\right)\right|\leq C. \end{array}$$

The solution of (24)–(26) can be expressed as

(29) $$\begin{array}{c} u^{\prime }\left(t\right)=u^{\prime }\left(0\right)e^{-(1/\varepsilon )\int_{0}^{t}a(s)ds}+\frac{1}{\varepsilon }\int_{0}^{t}F\left(s\right)e^{-(1/\varepsilon )\int_{s}^{t}a(\xi )d\xi }ds, \end{array}$$

and taking into account (26), we have

(30) $$\begin{array}{c} u^{\prime }\left(0\right)={\left(1-e^{-(1/\varepsilon )\int_{0}^{T}a(s)ds}\right)}^{-1}\frac{1}{\varepsilon }\int_{0}^{T}F\left(s\right)e^{-(1/\varepsilon )\int_{s}^{T}a(\xi )d\xi }ds. \end{array}$$

Thus we get

(31) $$\begin{array}{c} \left|u^{\prime }\left(0\right)\right|\leq \frac{C\alpha^{-1}\left(1-e^{-\alpha T/\varepsilon }\right)}{1-e^{-a^{*}T/\varepsilon }}\leq C\alpha^{-1}. \end{array}$$

The relation (29) along with (31) leads to (23) for *k* = 1 immediately.

Next for *k* = 2, from (1) we have

(32) $$\begin{array}{c} \left|u^{\prime \prime }\left(0\right)\right|=\frac{1}{\varepsilon }\left|f\left(0\right)-b\left(0\right)u\left(0\right)-a\left(0\right)u^{\prime }\left(0\right)\right|\leq \frac{C}{\varepsilon }. \end{array}$$

Differentiating now (1), we obtain

(33) $$\begin{array}{c} \varepsilon u^{\prime \prime \prime }+a\left(t\right)u^{\prime \prime }=f^{\prime }\left(t\right)-b^{\prime }\left(t\right)u-b\left(t\right)u^{\prime }-a^{\prime }\left(t\right)u\equiv \varphi \left(t\right). \end{array}$$

Under the smoothness conditions on data functions and boundness of *u*(*t*) and *u*′(*t*), we deduce evidently

(34) $$\begin{array}{c} \left|\varphi \left(t\right)\right|\leq C. \end{array}$$

The solution of (33) is

(35) $$\begin{array}{c} u^{\prime \prime }\left(t\right)=u^{\prime \prime }\left(0\right)e^{-(1/\varepsilon )\int_{0}^{t}a(s)ds}+\frac{1}{\varepsilon }\int_{0}^{t}\varphi \left(s\right)e^{-(1/\varepsilon )\int_{s}^{t}a(\xi )d\xi }ds. \end{array}$$

The validity of (23) for *k* = 2 now easily can be seen by using (32)–(34) in (35).

## 3. Example

Consider the particular problem with

(36) $$\begin{array}{c} a\left(t\right)=4,\;b\left(t\right)=3,\;f\left(t\right)=3t,\;T=1. \end{array}$$

The solution of this problem is given by

(37) $$\begin{array}{c} u\left(t\right)=A_{1}e^{-(\left(2-\sqrt{4-3\varepsilon }\right)/\varepsilon )t}+A_{2}e^{-(\left(2+\sqrt{4-3\varepsilon }\right)/\varepsilon )t}+t-\frac{4}{3}, \end{array}$$

where

(38) $$\begin{array}{c} A_{1}=\frac{2+\sqrt{4-3\varepsilon }}{2\sqrt{4-3\varepsilon }\left(1-e^{-\left(2-\sqrt{4-3\varepsilon }\right)/\varepsilon }\right)},\\ A_{2}=\frac{\sqrt{4-3\varepsilon }-2}{2\sqrt{4-3\varepsilon }\left(1-e^{-\left(2+\sqrt{4-3\varepsilon }\right)/\varepsilon }\right)}. \end{array}$$

For the first derivative we have

(39) u′(t)=−324−3ε(1−e−(2−4−3ε)/ε)e−((2−4−3ε)/ε)t−324−3ε(1−e−(2+4−3ε)/ε)e−((2+4−3ε)/ε)t+1

from which it is clear that the first derivative of *u*(*t*) is uniformly bounded but has a boundary layer near *t* = 0 of thickness *O*(*ε*).

The second derivative

(40) u′′(t) =−3ε(2−4−3ε24−3ε(1−e−(2−4−3ε)/ε)e−((2−4−3ε)/ε)t    +2+4−3ε24−3ε(1−e−(2+4−3ε)/ε)e−((2+4−3ε)/ε)t)

is unbounded while *ε* values are tending to zero.

Therefore we observe here the accordance in our theoretical results described above.
